# The relation between game disorder and interruption during game is mediated by game craving

**DOI:** 10.3389/fpsyg.2025.1579016

**Published:** 2025-06-04

**Authors:** Yanmeng Bao, Hui Zhou, Fengji Geng, Yuzheng Hu

**Affiliations:** ^1^Department of Psychology and Behavioral Sciences, Zhejiang University, Hangzhou, China; ^2^The State Key Lab of Brain-Machine Intelligence, Zhejiang University, Hangzhou, China; ^3^Department of Curriculum and Learning Sciences, Zhejiang University, Hangzhou, China; ^4^Children’s Hospital, Zhejiang University School of Medicine, National Clinical Research Center for Child Health, Hangzhou, China; ^5^MOE Frontiers Science Center for Brain Science and Brain-Machine Integration, Zhejiang University, Hangzhou, China; ^6^Nanhu Brain-Computer Interface Institute, Hangzhou, China

**Keywords:** internet gaming disorder, gaming interruption, craving, emotional arousal, anger

## Abstract

The burgeoning user base and potential negative effects of excessive involvement in gaming, particularly Internet Gaming Disorder (IGD), demand significant attention. While existing research has explored the susceptibility of individuals with IGD to game-related stimuli, the question of why it is challenging for these individuals to disengage from gaming remains under-explored. Drawing parallels with the concept of interruption, we hypothesize that negative emotions triggered during gaming interruptions would drive individuals’ craving for the game and compelling them to continue playing, reinforcing the IGD cycle. In this study, 42 male ‘League of Legends’ players, aged 19 to 29, experienced controlled interruptions every 3 min during gaming and non-gaming control tasks. Our findings demonstrate that interruptions during gaming elicited significantly higher levels of anger and anxiety compared to the control tasks. Further, we found a positive correlation between the severity of IGD symptoms and the intensity of anger and anxiety induced by gaming interruptions. Additionally, our analysis suggests that craving partially mediates the relationship between anger arousal during gaming interruptions and IGD severity. These findings provide new insights into how emotional responses to gaming interruptions contribute to IGD, offering a novel perspective for future research and potential treatment approaches.

## Highlights

- Gaming interruptions trigger anger and anxiety
- Stronger emotional arousals associate with severer gaming disorder symptoms
- Gaming craving mediates the relationship between anger and gaming disorder severity

## 1 Introduction

Internet gaming disorder (IGD), recognized as an international health concern, is characterized by the frequent use of the internet to play video games to such an extent that it results in clinically significant psychological distress or impairment (APA, 2013). According to meta-analyses, the average prevalence for IGD was 2.47% (95% CI, 1.46 –4.16%) (Pan et al., 2020), and the pooled prevalence of IGD among adolescents was 4.6% (95% CI, 3.4–6.0%) (Fam, 2018). In International Classification of Diseases 11th Revision (ICD-11), IGD has been officially classified as a mental disorder (World Health Organization, 2019). This classification underscores the significance and necessity of understanding and addressing the risks associated with IGD.

A key factor in addiction behaviors, including IGD, is craving, defined as an intense desire for engaging in psychoactive substances or behaviors (Blumenthal and Gold, 2010). Previous studies indicate that craving, particularly when triggered by addiction-related cues, plays a crucial role in the development, maintenance, and relapse of addictive behaviors (Goudriaan et al., 2010). In the context of IGD, craving has been identified as a critical component (Brand et al., 2019). Individuals with IGD consistently report higher gaming cravings in daily life compared to their healthy counterparts (Dong et al., 2017). This association suggests that craving is a significant contributor to the persistence and relapse of compulsive gaming in individuals with IGD (Wegmann and Brand, 2018).

“Loss of control,” which has been widely discussed in previous existing literature, is another core symptom of IGD. Individuals with IGD typically exhibit a lack of control over gaming, including frequency, duration, onset, and termination (World Health Organization, 2019). Paradigms such as cue-induced craving and response inhibition are employed to demonstrate that individuals with IGD encounter challenges in cognitive control or controlling game-related cravings (Dong et al., 2010) and experience gaming craving symptom during gaming abstinence (Yen et al., 2022). Despite substantial evidence on cognitive control and game craving, the reason why individuals with IGD have difficulties in voluntarily stopping the game remains unclear. This is a crucial daily in the game can inform treatments to mitigate IGD symptoms and reduce game time.

Withdrawal symptoms, which manifest when an individual is forced to stop internet gaming and include irritability, anxiety, or sadness (APA, 2013), are a critical indicator of gaming addiction and can help to explain why individuals with IGD spend a lot of time on gaming. These affective symptoms specifically arise in instances where an individual is either unable to engage in gaming or is consciously attempting to abstain from playing (Petry et al., 2014). Besides, Ko (2014) suggests that withdrawal symptoms should manifest at least 3 h after an individual’s last gaming session and can be alleviated by resuming online gaming. Research found that individuals with IGD experience withdrawal-related affective, gaming urge symptoms and a higher heart rate during abstinence from gaming for several hours (Yen et al., 2022). Therefore, withdrawal symptoms only accounts for the necessity that addicted individuals feel to play games daily; it falls short in explaining the extensive amount of time these individuals devote to gaming.

Interruption, which refers to “an unexpected suspension of the behavioral performance of, and/or attention focus from, an ongoing work task (Puranik et al., 2020), is a concept which rarely debated in the field of game disorder. Some notable studies have investigated that interruption can create a tendency or urge to return to the unfinished task (Ovsiankina, 1928) and evoke anxiety and annoy (Bailey et al., 2001; Jett and George, 2003; Üsten and Sieben, 2023). Therefore, to explain why individuals with game disorder spend a lot of hours in gaming everyday, we hypothesized that they cannot stop gaming because when stopping the game, a negative emotion response, such as anger or anxiety is generated, driving individuals’ craving for the game and compelling them to continue playing, reinforcing the IGD cycle. It is significant to note that the emotion response triggered during gaming interruption is different from withdrawal. Specifically, emotion response emphasizes on emotional arousal in a fleeting moment after interruption from on-going game.

To understand what specific types of emotions are induced during interruption in game and how they contribute to IGD, we designed a gaming-interruption behavioral experiment and employed questionnaires to assess IGD severity. Acknowledging the widespread presence of anger and anxiety in gaming addiction and its frequent identification in interruption science, we hypothesize that, compared to control conditions, game disruptions will elicit stronger anger in individuals. Additionally, we suggest that the intensity of this anger correlates with the level of gaming addiction and the relation is mediated by game craving.

## 2 Method

### 2.1 Participants

Concerning gender differences, men were the majority among those diagnosed with gaming addiction, with Bonnaire and Baptista (2019) and Ferreira et al. (2021) reporting 78.4 and 73.8% of gaming addicts being male. Although there was no intention to exclude female players, all 42 participants of “League of legend” (LOL) were male when we recruited through online advertisement. All participants were right-handed and had normal or corrected-to-normal vision. All participants completed questionnaires (refer to section 2.2.2) immediately after the behavioral experiment. One participant was excluded due to failure to submit the scale information, and three participants were excluded due to program errors impeding their completion of the experimental task. The data from the remaining 38 participants (mean age = 22.95, SD = 2.30) were used for subsequent analysis.

### 2.2 Measures

#### 2.2.1 Interruption task

**Figure 1:**
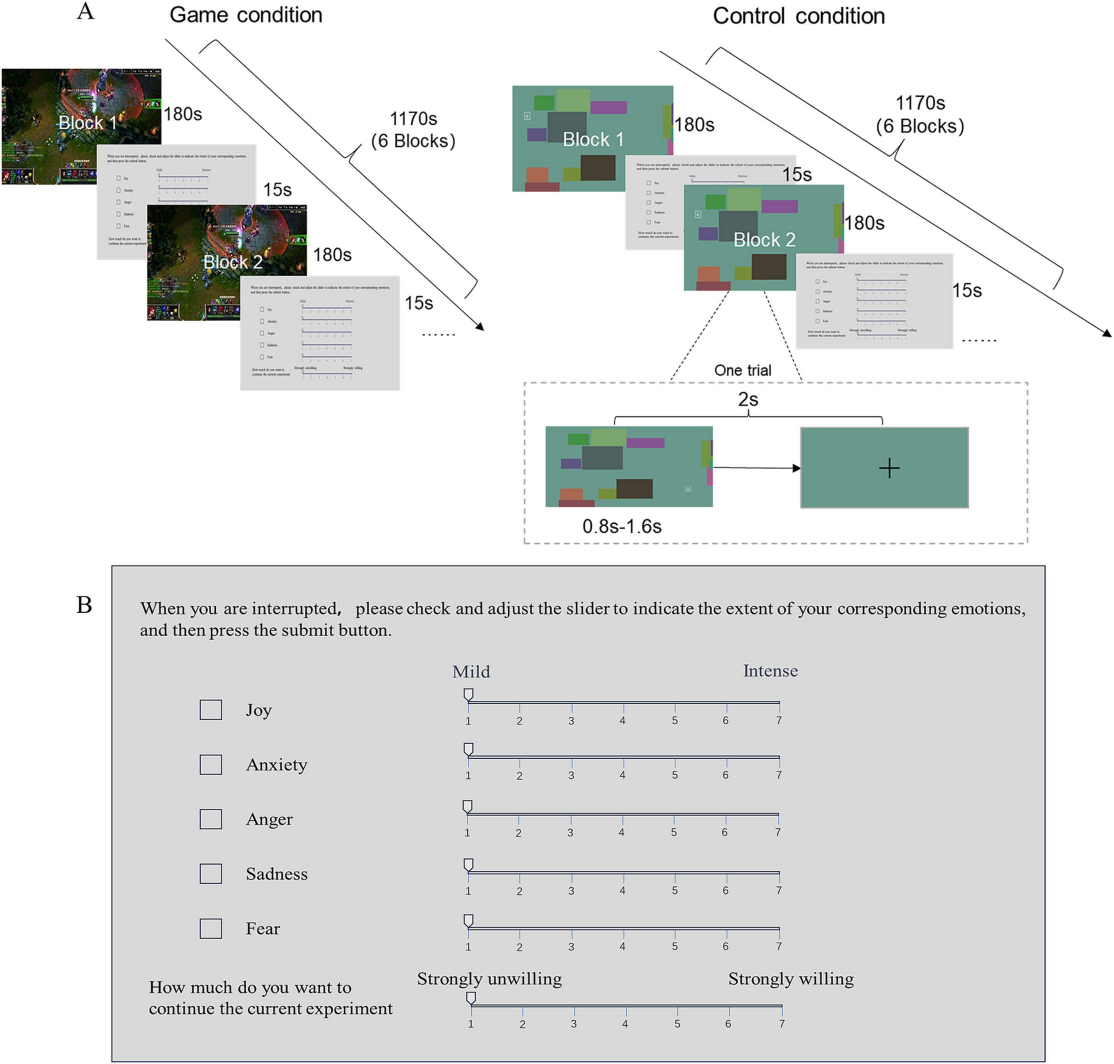
Estimation of emotional arousal in game condition and control condition. **(A)** Interruption task. In the game condition, participants are required to play League of Legends (an online game). In the control condition, participants are required to perform an identification task, which involves identifying the cues (Q, W, E, R, and dots) and responding by pressing the corresponding key or clicking with the mouse. Both tasks will be interrupted every 3 min to allow participants to self-rate their emotional arousals and craving for the current condition. **(B)** Emotion self-rate window. On the top of the window is the question asking “when the experiment is interrupted, how much of your corresponding emotions you have,” then from the top to the bottom are the emotions (joy, anxiety, anger, sadness, fear) and craving for the current experiment (rated on a scale of 1–7).

In this experiment, all participants were required to engage in a 20-min human-machine confrontation of LOL (Game condition) and a 20-min stimulus recognition task (control condition). Seventeen participants started with the Control condition, while 21 participants started with the Game condition (Figure 1A).

During the Game condition, participants engaged in a 5v5 LOL human-machine confrontation. The participant controlled his avatar using the Q, W, E, R keys on the keyboard and a mouse. The remaining characters were controlled by the LOL program. Participants were allowed to select a hero and adjust in-game parameters according to their preferred settings before experiment.

During the Control condition, the participants were required to perform an identification task. A single trial process is depicted in Figure 1A, where letter Q, W, E, R or dots were displayed on the screen at a random location with random color blocks as background. The Q, W, E, R were enclosed within hollow squares with side lengths equal to the diameter of the dots. The stimuli were presented for a duration of 0.8–1.6 s. Following participants’ key press or if they exceed the presentation time, a “+” cue will appear, indicating the end of the last stimulus and their readiness for the next stimulus. When the letter Q, W, E, or R appeared on the screen, participants were required to press the corresponding button within the stimulus presentation time. Alternatively, when dots appeared on the screen, participants should navigate the mouse over the dots and click on them using the left button within the stimulus presentation time. The screen background color was updated for each trial. This Control condition was designed to account for the motor responses during the game.

During both conditions, a gray background appeared every 3 min (6 times in each condition) to interrupt the process. Subsequently, a form window appeared for participants to report their emotions, including measures of joy, anxiety, anger, sadness, and fear, as well as their level of eagerness (craving) to continue playing under the current condition. All the measures were rated on a scale of 1–7 with a higher score indicating a higher level of emotional arousal or craving (Figure 1B). The interruption panel lasted for 15 s.

#### 2.2.2 Questionnaires

An online questionnaire was used to gather demographic information (i.e., age, gender, ethnicity, educational background). Gaming activity was assessed by measuring the average daily gaming hours, gaming frequency, and other questions related to gaming routine. The level of emotional arousals both in the interruption task and in daily game were also examined. The following standardized measures were utilized to characterize severity of IGD, craving for game, and general anxiety.

##### 2.2.2.1 Internet Gaming Disorder Scale–Short-Form (IGDS9)

The IGDS9-SF is a brief unidimensional psychometric test including a total of nine items reflecting all nine criteria for IGD outlined in the DSM-5 (Pontes and Griffiths, 2015), using a 5-point Likert reaction scale (ranging from “1 = never” to “5 = often”). The final score ranges from 9 to 45, with higher scores indicating higher severity of gaming disorder. Cronbach’s *α* for the IGDS9-SF was 0.9 and test–retest reliability ranged from 0.79 to 0.91. The Chinese version translated by Yam et al. (2019) was used in the present study.

##### 2.2.2.2 DSM-5 criteria for internet gaming disorder

The criteria including 9 self-report items to assess the DSM-5 IGD classification (APA, 2013). The items, which were developed by Petry et al.’s (2014) through an international consensus statement on measuring symptoms associated with IGD. These symptoms include preoccupation, tolerance, withdrawal, unsuccessful attempts to limit gaming, deceit, loss of interest in other activities, using despite knowledge of harm, using for escape or relief, and harm.

##### 2.2.2.3 Questionnaire on game urges-brief (QGU-B)

It is a self-reported craving scale on gaming. The questionnaire was adapted from the Questionnaire of Smoking Urges (QSU-B) (Cox et al., 2001) and consists of 10 items which scores from 1 to7 to represent gaming craving. The internal reliability of the scale was 0.99, and test–retest reliability was 0.96 (Ko et al., 2013).

##### 2.2.2.4 Generalized Anxiety Disorder 7(GAD-7)

The Generalized Anxiety Disorder-7 (GAD-7; Spitzer et al., 2006) was developed to identify probable cases of generalized anxiety disorder (GAD) and to assess symptom severity. The internal consistency of the GAD-7 was excellent (Cronbach *α* = 0.92). Test–retest reliability was also good (intraclass correlation = 0.83) (Spitzer et al., 2006).

### 2.3 Statistical analysis

The analyses proceeded in a structured sequence. First, we assessed the arousals of each emotion during both game and control condition. To compare participants’ emotional responses to interruptions between the game condition and control condition, we used both the mean and highest scores from the 6 interruptions. Second, we examined the correlation between levels of emotional arousal and IGD score, which was characterized by computing the mean of z-scores for DSM and IGDS9. Third, we explored whether the desire for gaming mediated the relationship between emotional arousals during interruptions and IGD severity, thereby connecting emotional arousals during interruptions to IGD through individual variations in the desire for gaming.

## 3 Results

### 3.1 Descriptive statistics of questionnaire data

**Figure 2:**
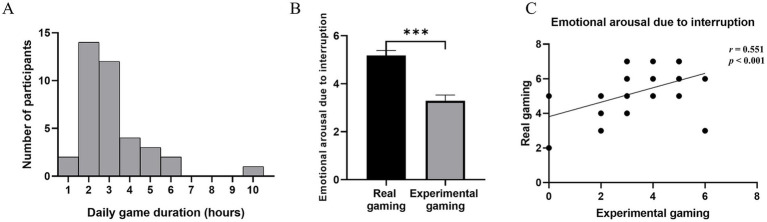
Estimation of daily game duration and emotional arousal level during experiment/real-life. **(A)** Daily game duration. The frequency distribution of the participants’ daily game duration. **(B)** Emotional arousal in game condition and real-life. Participants’ level of emotional arousal in a human-machine confrontation experiment was significantly lower than that during daily gaming. ***p < 0.001. **(C)** Correlation between emotional arousal in game condition and real-life. Participants’ level of emotional arousal due to interruption in experiment was significantly correlated with emotional arousal in real-life.

We first performed a descriptive statistical analysis of individuals’ IGD characteristics (Table 1). To investigate individuals’ daily gaming habits and the extent to which our experiment reflects their daily gaming behavior, we supplemented the scale described in section 2.2.2 with additional metrics. These metrics included participants’ gaming duration in real-life (Figure 2A) and self-rated scores of emotional arousals when interrupted during real gaming sessions and the current gaming experiment.

**Table 1:** Descriptive statistics of questionnaire data.

Variable	Min.	Max.	Mean	SD
Age	18.00	29.00	22.95	2.30
DSM-5	0.00	9.00	3.74	2.26
IGDS9	11.00	30.00	19.92	4.81
Game craving	10.00	53.00	27.87	11.86
GAD-7	7.00	26.00	12.97	4.49
Game duration	1.00	10.00	3.12	1.69
Emotional arousal (real game)	2.00	7.00	5.184	1.249
Emotional arousal (experiment)	0.00	6.00	3.290	1.505

### 3.2 Ecological validity examination

The self-ratings for emotional arousal ranged from 0 to 7, with higher scores indicating greater emotional arousal. The results revealed that the emotional arousal triggered by interruptions during daily gaming was significantly higher than that during the human-machine confrontation experiment (*t* = −8.397, *p* < 0.001) (Figure 2B). Spearman correlation analysis was conducted to examine the relationship between interruptions in the human-machine confrontation experiment and emotional self-evaluations during interruptions in daily gaming. This analysis revealed a significant positive correlation (*r* = 0.551, *p* < 0.001), indicating strong ecological validity of the experimental setup (Figure 2C).

### 3.3 Emotional arousals during the interruption

We conducted independent sample T-tests to examine whether emotional arousals would be influenced by the order of conditions (i.e., the Gaming condition first vs. the Control condition first). Our analysis revealed no significant differences in emotional arousals between participants performed the Gaming condition first and those who performed the Control condition first (*p*_min_ = 0.213). Consequently, experimental order was not considered as a confounding factor in further analyses. The average and maximum scores were used as two measures of emotional arousal during interruptions for each type of emotions. The maximum intensity was considered for two main reasons: (1) the gaming was interrupted at six fixed time points, some moments would be critical while others not for the user; (2) the likelihood that stronger emotional responses to interruptions may have a greater propensity to trigger gaming behavior and could be more relevant to gaming disorder. The descriptive statistics of emotional arousal during interruptions are summarized in Table 2.

**Table 2:** Descriptive statistics of affective arousal during interruptions.

Affective category	Game condition				Control condition			
	Average-level		Maximum-level		Average-level		Maximum-level	
	Mean	SD	Mean	SD	Mean	SD	Mean	SD
Joy	0.238	0.535	0.895	1.956	0.817	1.083	1.816	1.971
Anxiety	2.471	1.524	4.447	2.063	1.276	1.451	2.421	2.022
Anger	2.045	1.633	4.079	2.259	0.530	1.006	1.184	1.642
Sadness	0.280	0.698	0.737	1.446	0.225	0.569	0.684	1.435
Fear	0.289	0.761	0.816	1.768	0.056	0.249	0.237	0.883
Craving	5.476	1.199	6.395	0.755	3.422	1.631	4.711	1.738

**Figure 3:**
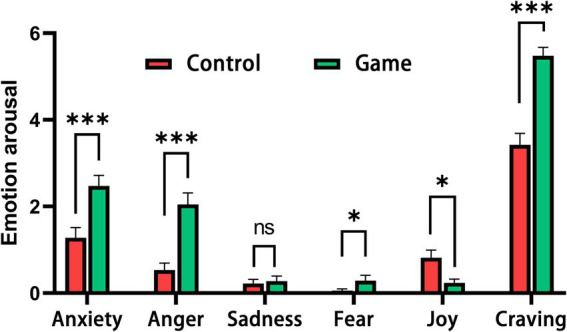
Estimation of emotional arousal in game and control conditions. The bar plots show the mean arousal level of each affective response in both conditions. *p < 0.05, ***p < 0.001.

The paired-sample *T*-test revealed significant differences in affective responses during the game condition compared to the control condition (Figure 3). Specifically, anxiety (mean = 2.472, SD = 1.524), anger (mean = 2.045, SD = 1.633), and fear (mean = 0.289, SD = 0.761) scores were significantly higher during game interruptions than during control interruptions (anxiety: *t* = 4.062, *p* < 0.001, Cohen’ *d* = 0.804; anger: *t* = 5.679, *p* < 0.001, Cohen’ *d* = 1.117; fear: *t* = 2.410, *p* = 0.021, Cohen’ *d* = 0.410). In contrast, joy scores were significantly lower during game interruptions compared to control interruptions (*t* = −2.703, *p* = 0.011, Cohen’ *d* = −0.679), while sadness scores did not differ significantly (*t* = 0.567, *p* = 0.574, Cohen’ *d* = 0.087). Besides, participants’ craving was significantly higher in game condition (*t* = 7.297, *p* < 0.001, Cohen’ *d* = 1.435). All the results were examined through 10,000-iteration permutation test using Monte Carlo method (Supplementary Table S1). The difference of maximum emotional arousals between game and control condition is similar to mean score of emotional arousals (Supplementary Figure S1).

### 3.4 The relation between IGD severity and emotional arousal levels

**Figure 4:**
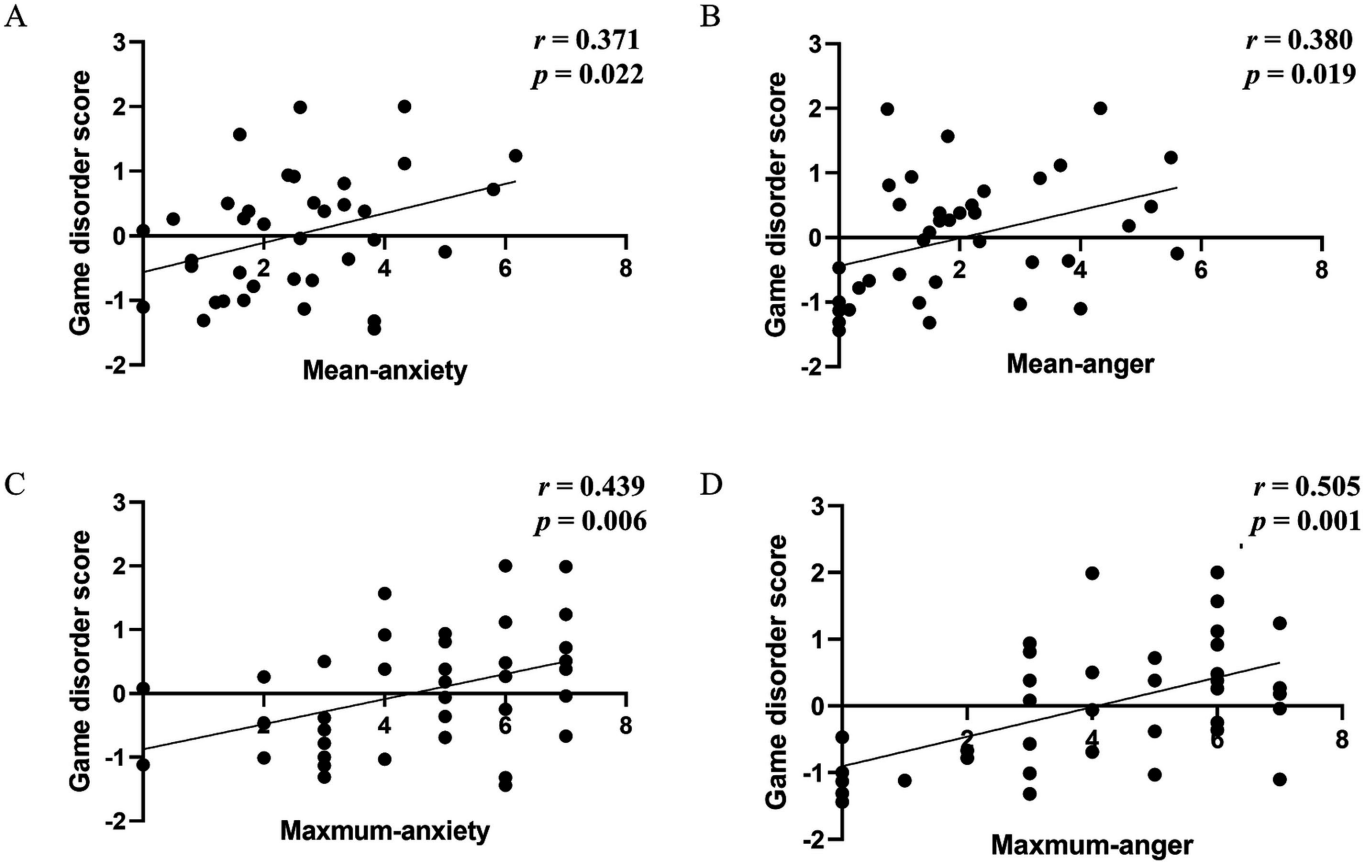
Correlation between game disorder severity and emotional arousal metrics in the game condition. **(A)** Pearson correlation between game disorder severity and mean-anxiety. **(B)** Pearson correlation between game disorder severity and mean-anger. **(C)** Spearman correlation between game disorder severity and maximum-anxiety. **(D)** Spearman correlation between game disorder severity and maximum-anger.

To quantify the level of gaming disorder, we transformed the DSM-5 scale scores (mean = 3.737, SD = 2.262) and the IGDS9 scale scores (mean = 19.921, SD = 4.812) into *Z*-scores and computed their average as the index of “game disorder severity.” Analyzing the relationship between gaming disorder severity and mean emotional arousal during interruptions in both control and game conditions revealed a noteworthy finding (Figure 4). Specifically, a significant positive correlation emerged between game disorder severity and the emotional arousals of anxiety and anger but only under the game condition (mean anxiety-disorder score: *r* = 0.371, *p* = 0.022; mean anger-disorder score: *r* = 0.380, *p* = 0.019, Figures 4A,B). This correlation was absent under the control condition. In-depth examination using partial correlation analysis, while controlling for age and generalized anxiety, indicated that anxiety and anger under gaming conditions displayed only marginally significant correlations with gaming disorder scores (mean anxiety-disorder score: *r* = 0.296, *p* = 0.075; mean anger-disorder score: *r* = 0.319, *p* = 0.054).

Since maximum emotional arousal intensity is ordinal data, Spearman’s correlation coefficient was used for the calculation. When employing the maximum emotional arousal values in Spearman rank correlation analyses, the findings closely resembled those obtained using the mean emotional arousal values: only the maximum emotional intensity of anxiety and anger during gaming conditions exhibited a significantly positive association with game disorder severity (anxiety maximum-disorder score; *r* = 0.439, *ρ* = 0.006; anger max-disorder score; *r* = 0.505, *ρ* < 0.001, Figures 4C,D). Additionally, when employing partial correlation analysis and controlling for age and generalized anxiety, anxiety and anger under gaming conditions still demonstrated a significant positive correlation with gaming disorder scores (maximum anxiety-disorder score: *r* = 0.408, *ρ* = 0.013; maximum anger-disorder score: *r* = 0.510, *ρ* = 0.001). The results of these correlations are also reported in the Supplementary Table S2.

### 3.5 Mediating role of game urges

Drawing from the theory of emotional motivation, it is postulated that higher levels of negative emotions can lead to increase in gaming activity, potentially plunging gamers into a state of game addiction through the desire for gaming. In line with this hypothesis, the study posits that the level of cravings for gaming serves as a mediator in the connection between the extent of emotional arousal during game interruptions and the degree of game addiction.

**Figure 5:**
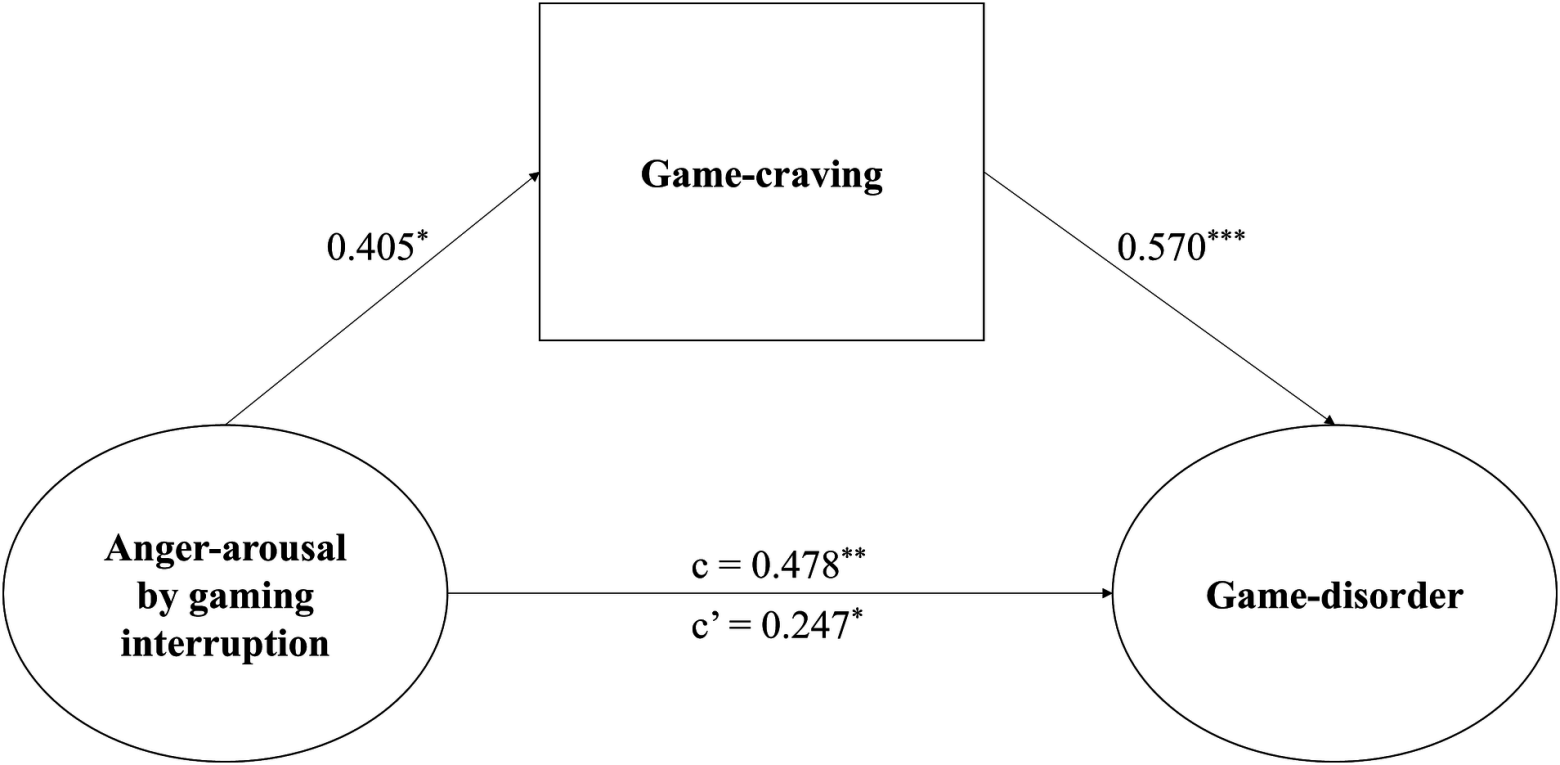
Mediation analyses demonstrated indirect mediation of craving on the relationship between interruption induced anger and game disorder severity. The total effect is indicated by path c, the direct effect by path c′, and the indirect effect is given by the product of path a and path b. The indirect effect was significant in the reported mediation. *p < 0.05, **p < 0.01, and ***p < 0.001.

In this model, the independent variables comprise the maximum levels of anger and anxiety during game interruptions. The mediating variable is the self-reported craving for game measured by QGU-B (measuring game desire right after the experiment), while the dependent variable is the game disorder severity. Additionally, the participants’ ages and level of generalized anxiety serves as control variables. Following the analytical procedures proposed by Preacher and Hayes (2008) and Baron and Kenny (1986), a bootstrap mediation effect test was performed using SPSS 22.0 PROCESSV3.4.1, involving 5,000 samples. The results indicated that only the model’s mediation effect related to anger during game interruptions was significant. The bootstrapped indirect effect was (0.405) × (0.570) = 0.231, and the 95% confidence interval ranged from 0.031 to 0.179. Thus, the indirect effect was statistically significant (*p* < 0.001) (Figure 5). They demonstrate that the mediating impact of game craving on anger after interruptions and game addiction was partial, with an effect ratio of 0.483. Conversely, mediation analysis did not reveal any significant mediation effect of daily game craving on the relationship between maximum anxiety during game interruption and game disorder severity (95% confidence interval ranged from −0.032 to 0.151).

## 4 Discussion

The current study revealed that levels of anger and anxiety arousal during interruptions in game were significantly higher compared to the control condition. Furthermore, levels of anger and anxiety arousal during game interruptions exhibited a robust positive correlation with the severity of IGD. Specifically, individuals with more severe symptoms of IGD exhibited heightened anger and anxiety when interrupted during gameplay. However, the relationship was not observed in control condition. In addition, the relationship between maximum anger arousal during interruptions and the severity of IGD was mediated by the craving of gaming.

Our results advance our knowledge on the role of emotion in IGD by showing the relationship between individuals’ emotional arousal during game interruptions and real-life IGD. This relation remained consistent across both mean arousal and maximum arousal, although subtle differences emerged, suggesting variations in the impact of negative emotional arousal on IGD in real-life. Additionally, our mediation analysis results demonstrated that maximum anger arousal during interruptions predicts individual differences in IGD through the mediating influence of game urges. These findings offer a novel insight into how game craving and negative emotion response during gameplay contribute to IGD.

The emotional response during interruption appears similar to withdrawal symptoms and both occur when the game is not available. However, these two concepts differ. Withdrawal symptoms pertain to symptoms that arise when one is unable to initiate gaming and/or deliberately attempting to stop gaming. It is suggested to be defined as symptoms that occur at least 3 h after the most recent gaming activity and can be relieved by further online gaming activity (Ko, 2014). It should be distinguished from an individual’s immediate reaction to stop a game (Petry et al., 2014). While the discussion around long-term withdrawal symptoms as a criterion for IGD has been extensive (Kaptsis et al., 2016), the immediate emotional reaction to ceasing gameplay has rarely been discussed. Our study demonstrates that negative emotional arousal, such as anger and anxiety, is not limited to long-term withdrawal, measured in terms of days, but can also occur during acute interruption, during game. Furthermore, we reveal that individuals with more severe symptoms of IGD exhibit greater anger and anxiety during interruptions in game. Our findings align with previously studies in interruption science which shows that interruption can evoke anger and anxiety (Bailey et al., 2001; Jett and George, 2003).

Additionally, our study sheds light on how the negative emotions such as anger and anxiety can manifest in daily life. Specifically, individuals with IGD struggle to stop gaming as negative emotions triggers anger and anxiety. Moreover, when interrupted by others such as parents, individuals with IGD would experience anger and anxiety, leading to conflicts in their family or school environments. This exposure to negative emotions may contribute to their mood-related psycho-pathological symptoms. Meanwhile, evidence from emotion regulation study also supports our findings. Individuals with IGD are found to have difficulties in emotion regulation (Lin et al., 2020), which may make it challenging for them to manage their negative emotions when interrupted during gameplay. Nevertheless, the relationship between anger arousal during game interruption and emotion regulation requires further investigation.

Additionally, our study indicated that game craving mediated the relationships between maximum anger arousal during game interruptions and IGD. While both mean and maximum of anger and anxiety arousal during interruptions in game were associated with IGD, only maximum anger arousal exhibited a relationship with game craving. Specifically, higher maximum anger arousal was linked to a stronger urge to play the game. This suggests that only maximum anger arousal during interruptions in game affect IGD through game craving, consistent with prior research findings that irritability is a mood-related psycho pathological symptom which is associated with IGD (Ko, 2014; Ko et al., 2012). This relationship indicates that maximum emotional arousal during interruptions may have a greater propensity to trigger gaming behavior and could be more relevant to gaming disorder. Furthermore, our findings suggest that anger may be the pivotal emotion driving individuals to persist in playing games.

The cue-induced reactivity paradigm (Ko et al., 2009) are widely used in previous IGD studies. In these studies, participants are triggered by game-related cue rather than real game. To our knowledge, this is the first study to apply real-game in an experiment setting to investigate the relationship between emotional arousal and IGD. Although interrupting positive experiences that leads to negative emotions may be a common mechanism of emotion change, we found that anger and anxiety arousals triggered by interruption of gaming, but not the control task, were related with severity of IGD symptoms. These results demonstrated that real-game in experimental setting is a viable approach for studying the characteristics of individuals with IGD. Besides, our findings indicated that emotional arousals during interruptions in game can be employed to understand individual differences on IGD.

Simultaneously, our results revealed that anger during game interruptions has an impact on IGD through its influence on game craving. It suggests that in the treatment of individuals with IGD, it is crucial to employ emotion regulation to preventing a reinforcing cycle of negative emotions. For example, mindfulness meditation, a therapeutic approach that assists individuals in directing their attention to emotional awareness without judgment (Alfonso et al., 2011), and has demonstrated beneficial effects in treating various psychiatric disorders, including addiction, can also be applied in IGD. Mindfulness aids individuals in accepting negative emotions triggered by interruptions during the game, viewing emotions as temporary mental events (Nyklíček, 2011), rather than persisting in playing games. Moreover, given the negative emotional arousal during gaming, applying the treatment during interruptions can be more beneficial.

Some limitations in our study need to be acknowledged. Firstly, our sample size is relatively small and we exclusively examine the emotional arousal level in the game League of Legends. The incorporation of additional external validation would add strong support to our findings. Secondly, we exclusively assess emotional arousal in male players, and the impact of sex differences on emotional arousal during interruption in game remains unknown. Thirdly, our study focuses on momentary expressions of IGD-related symptoms, not clinically diagnosed Internet Gaming Disorder. Fourthly, the experimental design introduces interruptions at fixed 3-min intervals. However, in real-life gaming scenarios, interruptions are often sudden, unpredictable, and context-sensitive. Lastly, we cannot predict the events that occurred in the game during interruptions in the gaming task. Although we utilize both mean and maximum arousal levels to indicate emotion, the impact of the game process on emotional arousal is still persist. Thus, more work is needed to replicate our thinking, employing alternative method to estimate an individual’s emotional arousal in game.

## 5 Conclusion

The present study demonstrated relationships between emotional arousal and IGD and supported the hypothesis that craving may be relevant in explaining the relationship between IGD and negative emotional arousal. We found that anger and anxiety aroused higher during game interruption compared to control task. Besides, individuals with more severe symptoms of IGD exhibited heightened anger and anxiety when interrupted during gameplay. In the mediation path model examined, craving partially mediated the association between anger arousal during gaming interruptions and the severity of IGD. The results suggest that anger might serve as the primary emotion motivating individuals to persist in playing games, reinforcing the cycle of IGD. Therefore, employing emotion regulation is crucial to preventing the reinforcing cycle of negative emotions in treatment of IGD.

## Data Availability

The original contributions presented in the study are included in the article/Supplementary material, further inquiries can be directed to the corresponding author.
