# Supplementary material for: The relation between game disorder and interruption during game is mediated by game craving
Source: Front Psychol. 2025 Jun 4;16:1579016. doi: 10.3389/fpsyg.2025.1579016 (PMC12174426; doi:10.3389/fpsyg.2025.1579016)
Supplement: Supplementary file 1 [file Supplementary_file_1.docx]

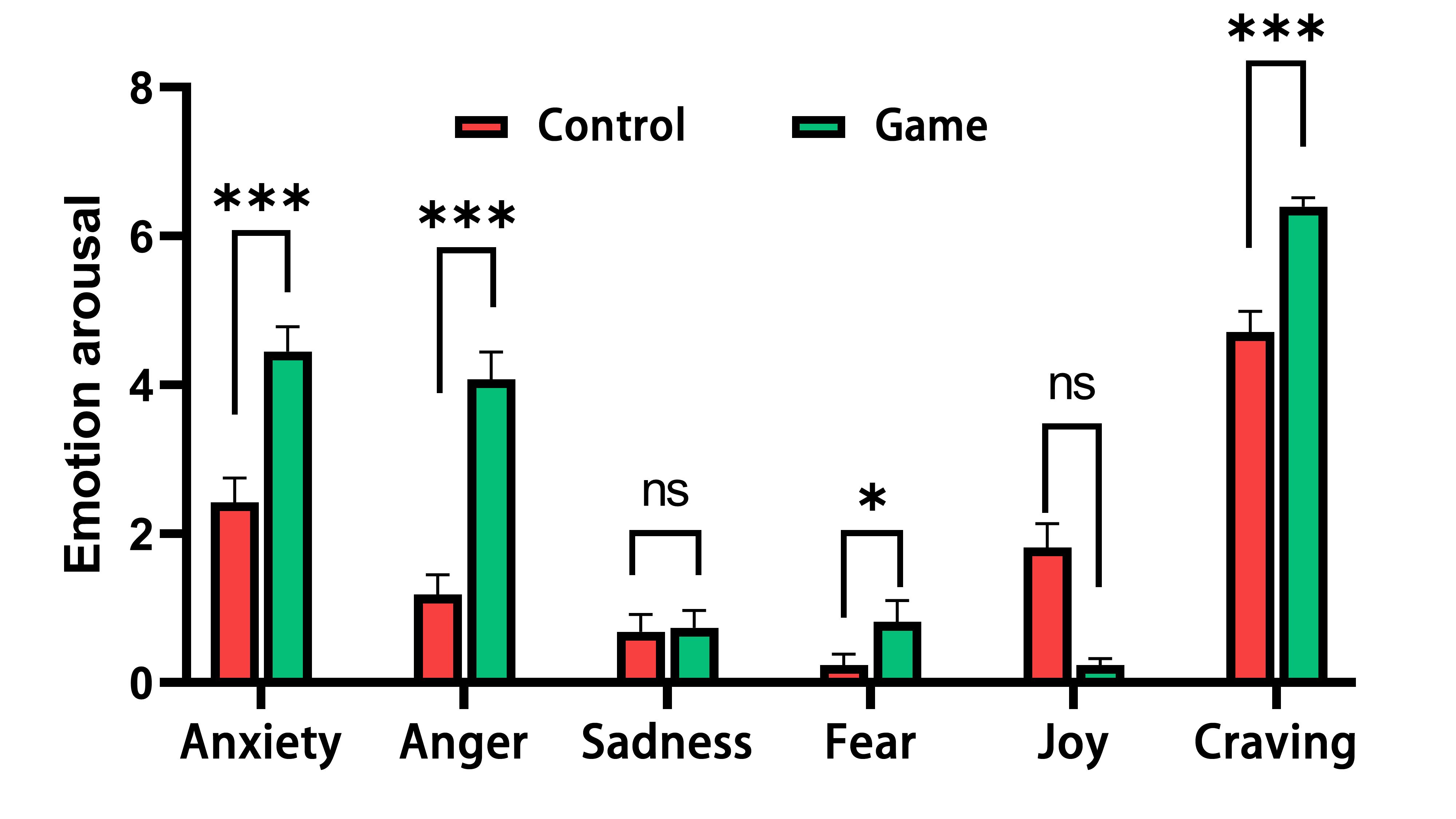


**Supplementary Figure 1. Estimation of max emotion arousal in Game and Control condition.** The histogram shows the average max emotion arousal of each emotion in both conditions and the results of significance test for the max arousal. **p* < 0.05, ****p* < 0.001.


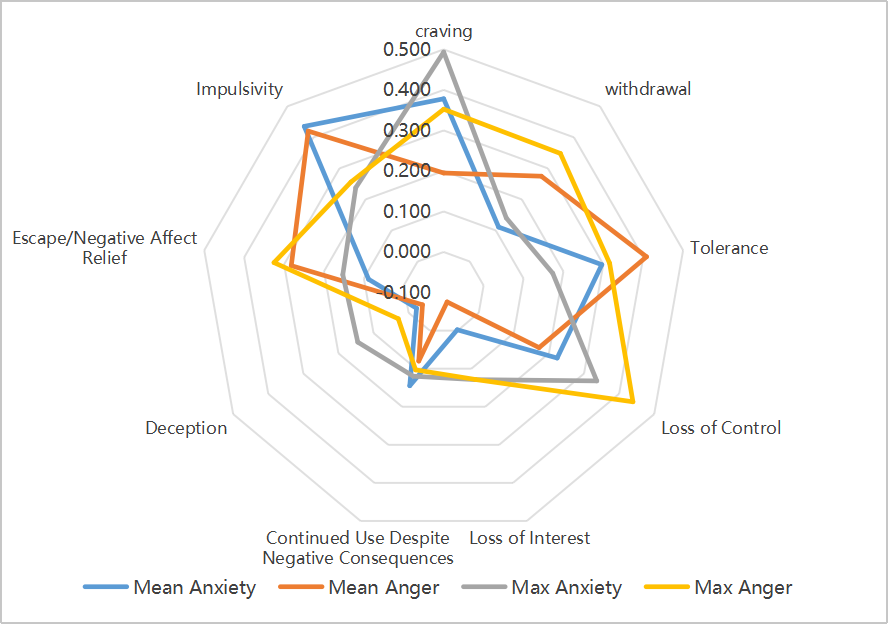


**Supplementary Figure 2. Correlations between DMS-5 symptoms and emotional arousals in the gaming-interruption task**


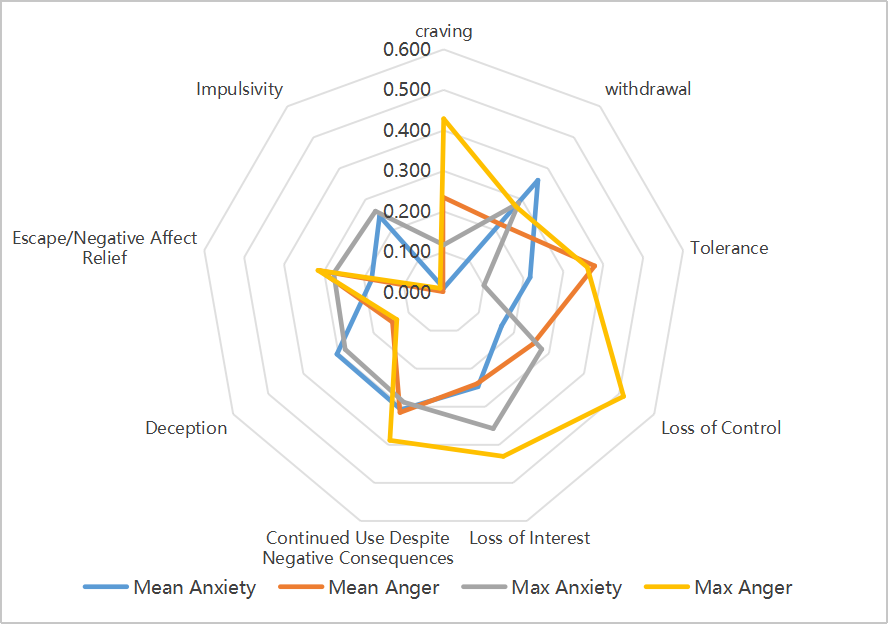


**Supplementary Figure 3. Correlations between IGDS9 symptoms and emotional arousals in the gaming-interruption task**


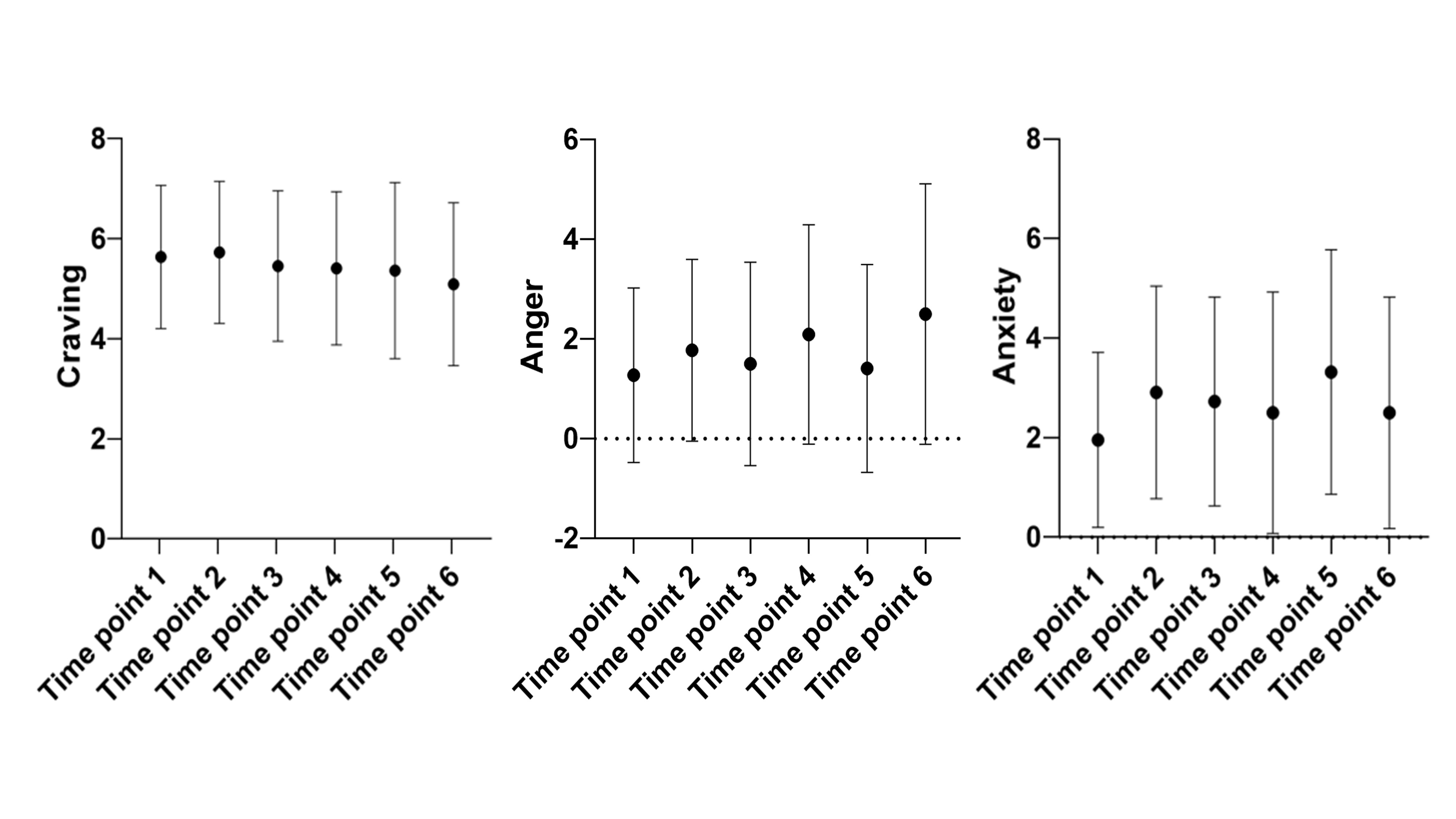


**Supplementary Figure 4. Craving, anger and anxiety levels at different time points.**

**Supplementary Table 1. Result of permutation test of emotion arousal difference between control condition and game condition.** Data are shown for *p* value of mean and max-mum emotion arousal (Joy, Anxiety, Anger, Sadness, Fear) difference in game condition and control condition.


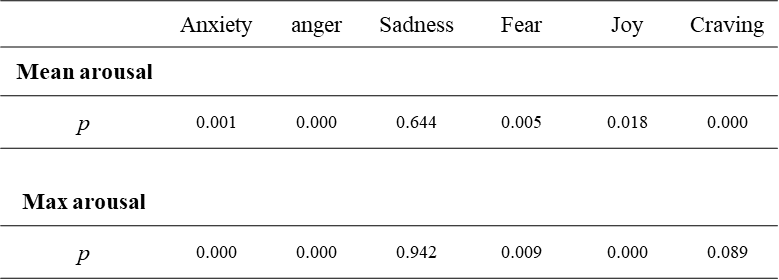


**Supplementary Table 2. Relationship between individual Emotion arousal metrics and Game disorder score.**

The Spearman correlations and Pearson correlations between Emotion arousal metrics and Game disorder are reported.


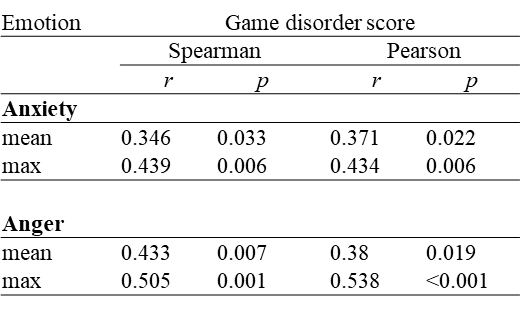


**Supplementary Table 3. Correlations between anger and anxiety arousals and IGD symptoms.**

|  |  | craving | withdrawal | Tolerance | Loss of Control | Loss of Interest | Continued Use Despite  Negative Consequences | Deception | Escape/Negative Affect Relief | Impulsivity |
| --- | --- | --- | --- | --- | --- | --- | --- | --- | --- | --- |
| DSM-5 | Mean Anxiety | 0.378* | 0.111 | 0.296 | 0.223 | -0.002 | 0.145 | -0.023 | 0.088 | 0.435** |
|  | Mean Anger | 0.195 | 0.275 | 0.408* | 0.172 | -0.075 | 0.08 | -0.04 | 0.282 | 0.42** |
|  | Max Anxiety | 0.493** | 0.14 | 0.173 | 0.336* | 0.129 | 0.12 | 0.145 | 0.153 | 0.238 |
|  | Max Anger | 0.352* | 0.348** | 0.316 | 0.439** | 0.128 | 0.103 | 0.029 | 0.325* | 0.256 |
| IGDS9 | Mean Anxiety | 0.011 | 0.362** | 0.217 | 0.165 | 0.247 | 0.308 | 0.304 | 0.181 | 0.246 |
|  | Mean Anger | 0.235 | 0.219 | 0.378* | 0.254 | 0.24 | 0.315 | 0.146 | 0.31 | 0.003 |
|  | Max Anxiety | 0.118 | 0.289 | 0.101 | 0.28 | 0.357* | 0.288 | 0.281 | 0.275 | 0.262 |
|  | Max Anger | 0.429** | 0.277 | 0.36* | 0.513** | 0.43** | 0.388* | 0.134 | 0.315 | 0.014 |

**p*<0.05, ***p*<0.01
